# Retraction Note: The α7 nicotinic acetylcholine receptor agonist GTS-21 improves bacterial clearance in mice by restoring hyperoxia-compromised macrophage function

**DOI:** 10.1186/s10020-020-00265-0

**Published:** 2020-12-30

**Authors:** Ravikumar A. Sitapara, Daniel J. Antoine, Lokesh Sharma, Vivek S. Patel, Charles R. Ashby, Samir Gorasiya, Huan Yang, Michelle Zur, Lin L. Mantell

**Affiliations:** 1 Department of Pharmaceutical Sciences, St. John’s University College of Pharmacy and Allied Health Professions, Health Sciences, 128 St. Albert Hall, 8000 Utopia Parkway, Queens, NY 11439 USA; 2 Medical Research Council Centre for Drug Safety Science, Department of Molecular and Clinical Pharmacology, University of Liverpool, Liverpool, UK; 3 Laboratory of Biomedical Science, Feinstein Institute for Medical Research, North Shore-LIJ Health System, Manhasset, NY USA; 4 Center for Inflammation and Immunology, Feinstein Institute for Medical Research, North Shore-LIJ Health System, Manhasset, NY USA; 5 Center for Heart and Lung Research, Feinstein Institute for Medical Research, North Shore-LIJ Health System, Manhasset, NY USA

## Retraction Note to: Mol Med (2014) 20:238–247 https://doi.org/10.2119/molmed.2013.00086

The Editors-in-Chief have retracted this article (Sitapara et al. 2014) following an investigation by the University of Liverpool. The investigation concluded that the mass spectra data contained in Fig. 4 demonstrate evidence of data manipulation, duplicate publication and figure fabrication and are thus unreliable. The mass spectra published in Fig. 4c are identical to mass-spectra published in Supplementary Fig. 2 from another publication (Wang et al. (2015)) and Fig. 3c in (Entezari et al. (2014)). In addition to this, there are inconsistencies between mass stated on the spectral peaks and the m/z (Da) value on the x-axis. There is also further possible figure duplication with some adjustment to the size of the peaks and labels. There is obvious similarity between spectra published in Fig. 4b in (Sitapara et al. 2014) and the spectra published in Fig. 3c in (Ju et al. 2014), with some alterations in peak size and mass labelling. There is further circumstantial evidence of research misconduct through inconsistencies in data produced by specific mass-spectrometers as detailed in the figure. The co-authors of the article were found by the investigation not to be complicit in any research misconduct, and they have been invited to resubmit a revised version of the manuscript for further peer review. More information on the university's investigation can be found on the university website (Further update on research misconduct investigation 2020).

All authors agree to this retraction.
